# News and Miscellany

**Published:** 1915-04

**Authors:** 


### News and Miscellany.

### A BRIEF REPORT OF FIFTH DISTRICT MEDICAL ASSOCIATION.

All those fortunates who attended the Fifth District Medical Association which convened in San Antonio, March 15 and 16, declared it to be the best and most harmonious of meetings. The papers were most helpful, and were all of a very high order, and were well received.

Dr. G. H. Moody of the Moody Sanitarium entertained the members with a buffet supper on Tuesday night, which was a perfectly delightful affair. The menu was splendid, and Dr. and Mrs. Moody made everyone welcome. While the affair was very elaborate it was very informal. But that was to be expected, for the Moodys are noted for their beautiful and enjoyable entertainments.

Dr. Charles Venable of the Lee Surgical Hospital entertained

Wednesday at noon with a unique luncheon. The weather was very disagreeable outside, rainy and cold, but the doctors were all there, and the striking contrast within warmed the hearts of all those present to Dr. Venable. The affair was informal and there was such an abundance of good things that everyone had all they could eat and drink. The get-together spirit was never demonstrated more than at this excellent luncheon.

The JOURNAL is glad to note that the doctors are coming to realize that the social features of their meetings are of great importance. The social gatherings are the doctors' play time, and no other class of men have greater need for play than the busy practitioner. At these sessions he forgets his duties for the moment, and finds time to meet his colleagues and they become better acquainted. These opportunities for the close personal touch adds much to the interest of the meeting.

We have just received a very handsome booklet from Dr. Edward C. Register of the *Charlotte Medical Journal.* The title of the booklet is "A Few Facts About the *Charlotte Medical Journal.*" It is beautifully gotten up, the typography is faultless and the handsome illustrations make one feel as though they had been privileged to visit in this sanctum sanctorium.

The booklet gives an account of the establishment of the *Journal,* its advertising rates, size of pages and its policy, along with an interesting account of the different departments and the field of thought covered by each.

The booklet is interesting and attractive and proves conclusively that nothing succeeds like success. It is a brief history of medical journalism in North Carolina for the past twenty-five years, and we are sure Dr. Register will be glad to send it to anyone who wishes to see it.

### A WOMAN'S NUMBER.

The May issue of the *Medical Review of Reviews* is to be a woman's number. All the articles contributed will be from the pens of women physicians whose work has achieved national importance. With the growth of the feminist movement the economic position of women has attracted universal attention. As medicine was practically the first profession open to women, it is only proper at this time to consider whether their entrance into the medical profession has been of benefit.

In order that women may present testimony by which they should be judged it has been deemed advisable to give them an entire issue to present the evidence of the value of their accomplishments.

In the laboratory, in the hospital, in institutions, at the bedside, and in public service, women physicians have performed a valuable function. As a tribute to their earnestness, enthusiasm, modesty, energy, perseverence, and scientific acumen, the May number of the *Medical Review of Reviews* will be dedicated to the women physicians of America.

THE RADIUM SITUATION IN AMERICA.

Dr. H. A. Kelly of Baltimore is one of the pioneer scientific workers in radium therapy in America. He owns more radium than any other individual in America, having between one and two grams of the element. Feeling the necessity of having more radium, Dr. Kelly, together with several others, organized the National Radium Institute, and he was made president of the corporation. This institution hoped, by an ingenious scheme, to secure seven grams of radium bromide for the expenditure of about $150,000. The Bureau of Mines experts were to develop a process—supervise the extraction of the radium, etc., from the ore, and if more than seven grams was obtained, this would go to the government. As a result, efforts were made to smother independent radium production, and to that end bills were introduced for the apparent purpose of conserving the radium deposits by withdrawing from entry all public lands containing this element, and by authorizing the Bureau of Mines to undertake the production of radium.

Chas. H. Viol, Ph. D., has published an article under the above caption relative to the production of radium in America, showing briefly by the following, that:

First. The present production of radium in the United States is more than adequate to supply the demand in this country.

Second. The selling price of radium is not exorbitant, and the prospects are that the price will rise in time rather than fall.

Third. The United States Bureau of Mines has given absolutely no proof whatever that the process for radium extraction said to have been devised by the experts in that department is at all successful in working upon a large scale the low grade carnotite ore which is at present the visible future of radium in this country.

Fourth. Should the government authorize the Bureau of Mines to go into the radium business on the scale contemplated in the acts under consideration, private parties and hospitals would not be able to secure such radium at any price until the National Radium Institute and the government hospitals had been supplied, and to secure such radium would involve waiting about five years.

Fifth. Should the Bureau of Mines be authorized to undertake to produce radium the result would be to practically confirm the "National Radium Institute" Company in a monopoly of radium for the therapeutic purposes for years to come, since the government hospitals may each only treat ten patients who are not government employes, and it is evidently the object of the radium legislation to try to make the production a government monopoly, forcing independent producers of radium to seek a market elsewhere, outside of the United States, or go out of business.

---

### Review of "Guiding Principles in Surgical Practice."

BY DR. FREDERICK EMIL NEEF.

This book should be of inestimable worth to the busy practitioner as being one from which he can readily glean the fundamental principles of surgery. The admirable arrangement, marginal headings and brief but accurate text outlining the preparation of patients, the conduct of the surgeon, assistants and nurses in preparation during and after operations, after treatment, dressings and anesthesia make it an invaluable asset to the profession.

---

WANTED—Set of Keen's Surgery. Please state number of volumes, condition and price of same. Address TEXAS MEDICAL JOURNAL, Austin, Texas.

---

### The Danger of Delay in Cancer.

Thousands of lives now needlessly sacrificed to cancer could be saved if the patient would go to the surgeon as promptly as does the average person attacked by appendicitis. Nor is there any reason why the cancer patient should not seek this, the only safe treatment, with the same high degree of confidence in the outcome that is now common among those suffering from the other more fashionable disease. Unfortunately, the evidence is only too clear that a different attitude toward cancer prevails and occasions many preventable deaths. The almost superstitious dread of the disease and unwillingness to admit its existence or to seek medical advice in time are well known and difficult obstacles to progress in its control. Proof of this fatal neglect is found in the experience of a prominent surgeon who recently studied his case records in order to obtain definite information as to the delay in the average case. Of 65 recent patients 35 were men and 30 were women. Further study of these 65 cases showed that after the first discovery of suspicious symptoms the men had waited an average of 12.2 months before consulting the doctor, and the women had waited, on the average, 11.9 months, practically a year's delay in all cases. Many other surgeons could produce very similar records. Winter of Koenigsberg, Prussia, the pioneer in the education of the public in regard to cancer, examined the records of 1062 operable cases and showed that 87 per cent of these patients could and should have applied for treatment much earlier, when they would have had a far higher chance of recovery than was actually the case.

To the delay when the symptoms are manifest must be added the previous indefinite period after the beginning of the disease and before the patient realizes the trouble. This period can be shortened by education. Fortunately the symptoms of cancer are present quite early and can usually be recognized if the patient understands their importance. In too many instances, however, the disease is not suspected until the symptoms are pronounced or until there is a tumor of considerable size. If we assume that this period averages six months, and then add the year's delay for which the patient is responsible, we find that the average patient does not seek advice until at least a year and a half after the onset of cancer. This precious time thrown away means, if not a fatal outcome, at least a serious instead of a minor operation.

In the present state of our knowledge of malignant disease it cannot be too frequently emphasized that the hope of curing cancer is to be found in its earlier recognition and in prompt and competent surgical treatment. The unfortunate patient who, because of ignorance or unwarranted fear or the blandishments of quacks, hesitates to seek proper advice should realize that in this delay he or she is recklessly throwing away a splendid chance of cure.—*Press Service of American Society for Control of Cancer.*

---

### The Harrison Anti-Narcotic Law.

The Harrison anti-narcotic law, which went into effect March 1, is a Federal statute to control the sale of opium, morphine, heroin, cocaine and similar drugs. The law requires that the manufacturer of these drugs keep the revenue department informed as to the output of their plants and to whom and in what quantities they sell. The wholesaler is required to report the amounts of his purchases from the manufacturer, and to whom he sells. Physicians and dentists are required to use blanks furnished by the revenue department when they write prescriptions, and they are required to keep duplicate copies of all prescriptions for the inspection of the revenue officers, and druggists are required to number and file the prescriptions and keep them where the revenue officers can inspect them.

In other words, the law was framed so that the government can keep track of every ounce of the drugs mentioned, and no druggist or other person is permitted to sell such drugs except on the prescription of a physician. Any person who violates or fails to comply with the provisions of the law shall be liable to a fine of not more than $2000, or imprisonment for not more than five years, or both, in the discretion of the court.

